# Being away from home for cancer treatment: a qualitative study of patient experience and supportive care needs during radiation therapy

**DOI:** 10.1002/jmrs.578

**Published:** 2022-04-04

**Authors:** Vanessa Knibbs, Stephen Manley

**Affiliations:** ^1^ North Coast Cancer Institute Lismore New South Wales Australia

**Keywords:** Cancer, non‐clinical, nursing, patient care, radiation oncology, radiation therapist, research – qualitative, treatment

## Abstract

**Introduction:**

Supportive care needs (SCN) refer to support required by patients and their families to better cope with cancer. Many rural radiation therapy (RT) patients stay away from home for significant periods, which can lead to the negative effects of both social isolation and cultural disparity. They may demonstrate complex SCN. This study aimed to explore experiences of being away from home by considering patient perspectives of their own SCN. The objectives were to provide a deeper understanding of how these patients think and feel and present a foundation of patient‐centred insights for further research.

**Methods:**

Thirteen patients participated in semi‐structured interviews; all stayed away from home for RT at the North Coast Cancer Institute for >3 days a week for >3 weeks. The data were subject to interpretive phenomenological analysis: a thorough process of understanding and analysis that is accompanied by reflection to improve researcher transparency.

**Results:**

Two themes influenced patient experiences of their care: values and identity, and expectations. Patients discussed the value they place on rural life, community connections and health care and referred to information for managing expectations. SCN discussed fell into practical, physical and psycho‐social needs.

**Conclusions:**

Experiences of culturally appropriate patient‐centred supportive care improve control and confidence. Patient well‐being is influenced by compassionate, caring and respectful connections with others. Several practical ways of managing expectations and promoting the psycho‐social well‐being of patients are discussed, for example, tailored packing lists and easy access to green spaces. Future research can be shaped by lived experiences.

## Graphical abstract

This study explores radiation therapy patient experiences of being away from home; it considers patient perspectives of their own supportive care needs and identifies recurring themes. It provides a deeper understanding of how these patients think and feel and encourages the foundation of patient‐centred insights for further research.

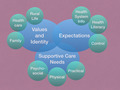

## Introduction

Supportive care needs (SCN) refer to the various forms of support required by patients and their families to better cope with cancer.^1^ Prior research has identified that needs tend to fall into five domains: physical and daily living, psychological, health system and information, patient care and support, and sexuality.^2^ As treatment complexity increases, so might these needs, leading to a risk of unmet SCN. This risk has been linked to a reduced patient quality of life and worsened emotional stress.^3^, ^4^ SCN have been researched for a range of cancer types and stages of diagnosis,^5^ treatment^6^, ^7^ and survivorship.^8^, ^9^

For the purpose of this study, ‘rural’ patients have been defined by the most recent model used by the Australian government to classify rurality for health workforce planning.^10^ All participants reside in a small, medium or large rural town area and travel more than 2 hours to get to the treatment centre and back home. A small body of evidence assessing the SCN of rural cancer patients suggests they have an increased risk of having unmet SCN.^1^, ^9^ Rural cancer patients have reported concerns about travelling for radiation therapy (RT), which can be expensive and inconvenient and lead to social isolation^6^, ^11^ and cultural disparity.^12^

A recent literature review of rural patients’ SCN found 10 out of 23 studies identified a negative impact of social isolation.^9^ The cancer experience frequently created significant psychological and emotional disruptions including the following: the lack of services rurally, difficulties with practical needs, uncertainty/fear around travelling and lack of privacy. A desire to discuss concerns with people in similar situations was commonly identified. Positive features of rural life were also recognised, including a sense of peace and a learned survivorship engendered by living in remote areas during other adverse life events. Tailored interventions deserve further research.

An in‐depth study examining the impact of travelling for RT of 19 patients and their carers from remote communities in Queensland was conducted in 2002.^6^ It highlighted significant disruption and stress on family life. The findings may have been diluted as they discounted patients recently diagnosed, and many carers felt too distressed to participate.

The prior research identifies that rural cancer patients often exhibit several unmet SCN, which are complex, dynamic and patient‐specific. The department this study was conducted at receives RT referrals from a vast geographical range including many rural and remote communities.^10^ Approximately, 20% have extended periods away from home to avoid long and expensive journeys. The current statewide Strategic Plan for RT recognises the significant social burden involved for these patients,^13^ and the recently published Community Engagement Framework^14^ demonstrates a drive to develop services based on local experiences/needs. Prior to conducting this study, a review of the three largest studies was conducted, presented and discussed at a departmental meeting (see Supporting Information, Appendix S1).

A lack of recent research exploring the lived experiences and needs of these patients was identified. Patients need to be directly consulted about their SCN – safely, ethically and constructively. The research question, ‘How do patients that stay away from home for radiation therapy treatment perceive and experience their supportive care needs?' aims to explore experiences of being away from home by considering patient perspectives of their own SCN. It is expected to provide a deeper understanding of how these patients think and feel, to present a foundation of patient‐centred insights for further research and discuss how to better meet the SCN of patients who stay away from home for treatment.

## Methodology

An interpretive phenomenological approach was taken; thus, the research was not about uncovering one truth but trying to understand the essence of the phenomenon by examining the views of those experiencing it. An extensive evaluation of the methodology can be found in Supporting Information, Appendix S2. This research gained approval from the North Coast NSW Human Research Ethics Committee.

### Methods

Semi‐structured interviews explored the phenomenon in great detail through open conversation, guided by the patients’ emerging viewpoints. An interview guide (see Supporting Information, Appendix S3) was developed to ensure commonly measured domains of SCN were discussed for all participants.^2^ The guide was reviewed by several health professionals to ensure professional validity. Interviews were conducted in an open‐ended way to encourage conversation flow and openness. Interviews took place face‐to‐face, confidentially and at least 2 weeks into their RT regimen.

### Sample

Purposeful typical‐case sampling was used: the researchers used prior knowledge about the purpose of the study so that participants could be appropriately identified and approached. Invited participants met the following criteria:

- Currently having RT treatment >3 weeks.
- Staying away from home but close to the treatment centre for >3 days a week.
- Home address located >2 hours round trip from the treatment centre.
- English‐speaking adults.

Sampling continued until information redundancy/saturation occurred, whereby analysis revealed no new emergent themes within the context of the research question. Data collection and analysis occurred simultaneously, so researchers recognised this conclusion. Data saturation originates from Grounded Theory methodology and has since gained acceptance within a range of qualitative approaches.^15^ Interviews were 20–90 minutes, with an average time of 55 minutes. The same researcher conducted the interviews and completed the data analysis. The research supervisor has experience in both RT research and leadership. Care was taken to minimise the risk of participants having a therapeutic misconception, that is, the decision to participate may affect their care, by ensuring the participants had not already met the researcher in a therapeutic role.

### Data analysis

Data were subject to interpretive phenomenological analysis (IPA), a rigorous three‐part process: naive understanding; structural analysis; and comprehensive understanding and reflection.

A journal was kept, and dialogue was conducted with the supervisor and health professional colleagues. This reflexivity gave the interpretation process credibility, transparency and aided the identification of potentially damaging preconceptions or bias.^16^ As with the nature of interpretive phenomenology, it is recognised that the chosen approach has been influenced by the researcher’s own experience, values and beliefs and that there are multiple socially constructed realities which are experienced differently by different people and influenced by context. The elements of research compliment each other in clear consensus; the interpretive phenomenological paradigm that frames the research question, the sampling and analysis methodology and the researchers own ideals.

The novice qualitative researcher used several published guides^17^ and checklists^18^ to ensure the methods used were sound and thorough. Table 1 assesses rigour and trustworthiness using a long‐standing framework^19^ and the recent criteria identified for allied health professionals.^16^ Supporting Information, Appendix S2 provides more details.

**Table 1:** Criteria for ensuring methodological consistency in qualitative research.

Criteria	Summary	Methods employed
Trustworthiness	Transferability, dependability and confirm‐ability of the research	Checking for understanding, repeating back any key points during the interview, reflective listening Asking participants to review the researcher’s notes to correct any misunderstandings Thick descriptions: conclusions drawn are given context wherever possible and superficial descriptions avoided Bracketing and a reflexive journal helped uncover researcher bias, motivation and interest Regular dialogue with the more experienced research supervisor allowed assumptions to be questioned and consensus made
Credibility	Confidence in the truth of the findings	Researcher has prolonged (9 years) experience as a radiation therapist working with patients from rural and remote areas Persistently conducted the interviews until a deeper understanding was gained from a range of patient experiences and perceptions Comprehensive accounts obtained from multiple independent participants Transparent coding process Peer debriefing was conducted
Rigour	Measure of research quality	Piloting interviews and interpretive phenomenological analysis (IPA) Using the same, evidence‐based, interview guide Similar interview environment for each participant Triangulating the data: collecting data from more than one participant and requesting regular feedback from supervisor and colleagues Rigorous method of IPA

## Results

### Participant details

Thirteen participants, all of who met the selection criteria, see Table 2 for more information.

**Table 2:** Participant details.

Gender	8 male 5 female
Treatment site	5 chest/thorax 4 head and/or neck 3 pelvis 1 skin of thorax
Location of staying away	10 stayed at Our House (subsidised accommodation) 2 stayed with family, friends 1 rented a unit nearby
Support while away	5 stayed away from home alone 8 had a family stay with them for some or all of the time away from home
Weekends (not reviewing RT treatment)	6 did not go home at the weekends 6 went home most weekends 1 went home both at weekends and 1 night during each week 9 drove themselves from home to the treatment centre location and back 4 had a family member drive them

#### Themes

**Figure 1:**
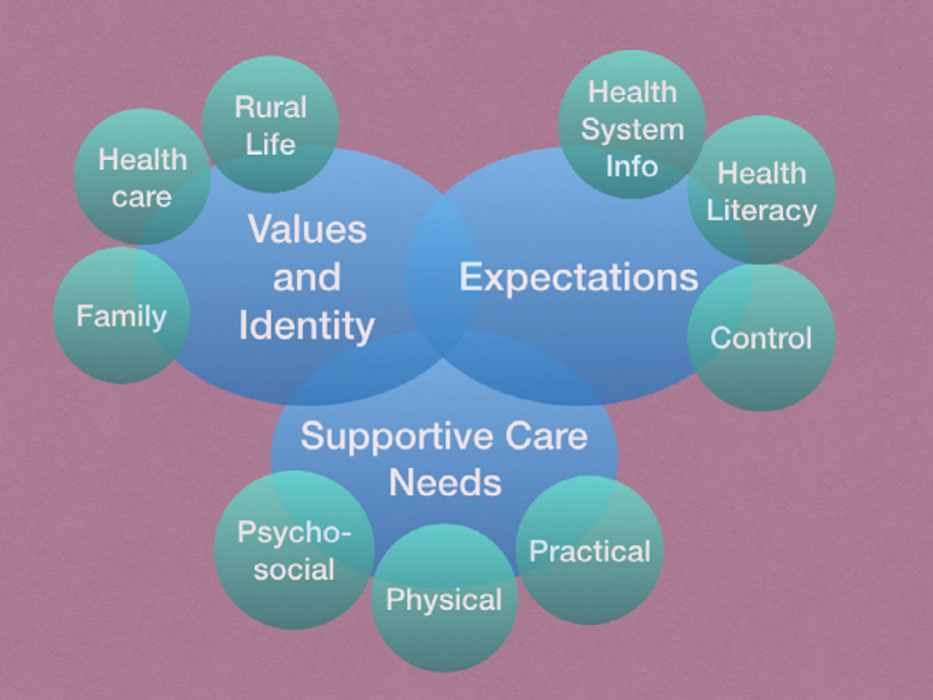
Summary of themes in the qualitative study of patient experience and supportive care needs during radiation therapy.

Interpretive phenomenological analysis uncovered two key themes which influenced patient experiences and perceptions of their care: values and identity, and expectations. The SCN discussed fell into three main themes: practical, physical and psycho‐social. These broad‐level themes emerged from rigorous data analysis, and they are summarised above in Figure 1 and demonstrated by quotes from the interviews as shown in Tables 3, 4, 5. Inevitably, some share characteristics or are linked in some way and are in no order of priority.

**Table 3:** Quotations for patient identity and values.

*Values and Identity:* Invaluable contextual information was gained from patient histories and significant life events. Patients talked about their personal ideology shifts, sources of current attitudes, resilience levels, coping strategies, levels of support and why they live rurally	
Summary	*Interview Quotes*
*Values of rural living:* Value was placed on small communities, yet privacy was more important for some One participant described how a tragic event had led to a recent shift in their community values	*‘You have to like small towns to be able to live in it. I love small communities, you’ve got that good neighbourhood watch and you have got people looking after you’* *‘I had a couple of nasty experiences with neighbours right on our doorstep… We have got freedom, it’s much freer than if you have someone close up against you’* *‘I am beginning to think sometimes it’s good not to be around people… I was very social and very community minded… but I had a really tragic thing happen in my life… It sounds awful… I didn’t want anyone to know this was happening, because they caught me early and I thought well let’s just get over this and get this thing done. Then one of the women rang me… of course being a small village now everyone knows. So that’s where I am at, it’s been a bit hard’*
*Valuing health care:* Some participants reflected deeply into their diagnosis and cancer journey, often placing high value on local cancer services	*‘Healthcare should be there for those that need it… I can even remember the nurses names, I think it must be because it’s a focal thing on my mind, what I’m here for’*
*Family values*: These were paramount for some, even if living rurally meant they did not often see their families Others accepted that undergoing cancer treatment away from home meant they needed to put themselves first	*‘I will just do everything in my power, I really want to be here until my grandson turns 21… I will do everything I can in my power to make that happen, if that means going for more treatment or having more tests then I will do it’* *‘My granddaughter is 15, going on 21. I feel that’s what is keeping me going’*
*Spirituality/Faith:* Some participants, who have faced serious illness in the past, talked about how faith or spirituality has got them through	*‘I’m fine, I’ll get through this… It was my attitude when I had cancer (before). I’m here for the long haul’* *‘A little gospel song we used to play… right back since 2014 to now, that song has meant God was with me. I used to laid up in the hospital and sing… all though my sickness I’ve been playing it and I still reckon that’s what’s got me to where I am. I’ve had a long battle’*
*Resilience:* Open‐ended questioning and active listening of patient’s stories and memories gave insight into their attributes of resilience, personal growth, coping strategies and levels of support	*‘I’ve always been very resilient. You could hit me with a bloody ton of bricks, they’ll just bounce off me, I just keep going’* *‘Well we had a pretty rough time. 18months of bad drought… and when that was nearly finished we had 5 bushfires… out of 6000 acres about 4500 got burnt… It wasn’t a good time – anyway it’s something you’ve gotta do, you’ve just gotta do it’*

**Table 4:** Quotations for expectations.

*Expectations*: As a patient staying away for treatment all participants talked about how their expectations have been managed at various stages (diagnosis, treatment and follow up). In cases where expectations did not appear to be managed well it was exacerbated by the fact that they live rurally and/or >100 km from the medical facility	
Summary	Interview Quotes
*Health system and information*: One participant requested more detailed information tailored to breast cancer and the DIBH technique for those who live far away from the treatment centre Another offered others advice for the DIBH technique. They both expressed a sense of misunderstanding and loss of control, exacerbated by living far away from the treatment centre	*‘I personally would have found useful something specific to breast cancer. The thing I’ve had trouble with was the breathing, if they’d given me info… specifically for breast cancer as it is a little different wherever you go’* *‘I think just say to practice your breathing lying down, because it’s totally different, that’s one thing that I learnt’*
*Building on past or others’ experiences:* Some had previous experience of cancer, others had talked to friends or family who had had similar experiences, so they could anticipate what to expect	*‘Three of (my friends) all said ‘look me radiation doesn’t hurt, so don’t be frightened of it… no two people are going to react the same. But something is being done so you have to respect that you are going to feel different in some way’* *‘But everyone is a bit different how it affects them… some people are full of doom and gloom and if you talk to the wrong fella it might make you not want to come’*
*Control:* One participant was unsure he wanted treatment at all, a major concern for him was staying away from home. This participant expressed relief that the doctor took the time to call his daughter. Support was delivered in a timely, professional and personal way to ensure the patient understood what to expect and felt in control especially when he had to spend 5 weeks away from home alone Another talked positively about being given a sense of control by the treatment staff	*‘I rang the nurse up here and told them that I don’t think I want to go… they get the doctor to give me a ring. I couldn’t understand some of what he was saying, my old brain couldn’t pick it up. He offered to ring my daughter and I said “would you?” and then when my daughter rang me back after she told me everything he talked about. She said “Dad stop that pig headed you got and just try it to see how you go?” and I told the doctor I would give it a go and he said “your wishes is my wishes”’* *‘Everybody has been really nice and they tell you what they are going to do, and they don’t do anything unless you say yes’*
*Managing expectations:* More than half talked about having their expectations managed well by medical professionals One participant that said he didn’t know what to expect did not have his accommodation arranged until the day before treatment started. He also stated that despite the 220 km round trip, he has never met the doctor and does not like telephone consultations One patient described how shocked they were at their first appointment. He lived so far away that he was consented for treatment, had his immobilisation mask made, and planning CT scan all on one day. Thus had no time to fully appreciate the mask‐making implications, spent 4 hours with staff and then had a 2.5 hour drive home	*‘They give me a fact sheet and showed me around a week before, we had a good chat and everything was in place before I come up here to start my treatment, it was good in that context’* *‘I came down here to see the radiation oncologist (RO), and he was so nice and explained everything and what they were going to do and I’ve had no problems with it… My breast care nurse she told me about the accommodation over the road and then the RO told me about it, we talked to the social worker and she booked us in’* *‘I prefer face‐to‐face appointments – my hearing is not the best… I’ve not met the radiation oncologist, he does exist doesn’t he?’* *‘I didn’t know what to expect when you made my mask. I wasn’t expecting something like that…I didn’t know what the treatment was going to be… Yeah it was alright but when you don’t expect that’*

**Table 5:** Quotations for themes of supportive care need.

*Psycho‐social Supportive Care Needs:* The psychological strains of having cancer treatment away from home were often raised. A few ideas were discussed on how to improve social and psychological support for patients who stay away from home	
Summary	*Interview Quotes*
*Being supported by the health care professionals:* Most participants praised the support they received from staff to help them get through. Some participants experienced confusion and anxiety following their diagnosis. Others talked about everything happening so quickly they must be ‘imagining’ it. Only one participant said psychological telephone support from a counsellor had helped him get through being away from home	*‘This came on suddenly, it’s been very stressful, but the lady (social worker) who helped us organise it here was wonderful, it would have been even harder without that’* *‘I was (worried) when I was first diagnosed as it came out of the blue… So I was rattled to start with but the more I saw other people and they reassured me. My doctor said if you’ve got to have breast cancer, then this is the best type to have, so I was reassured all the way. I’ve got over the “I’m going to be dead next week” thinking after a couple of weeks’* *‘The (staff) are all very very good, I cannot fault one of them. They ask if I am comfortable’* *‘Best staff here I’ve had for a long time. Very caring, very kind, very polite’*
*Having supportive loved ones:* Good social support and psychological well‐being were shown to be inextricably linked; those who had the close support of friends or family members while they were going through treatment away from home talked with more emotional stability about their experience Most participants recognised the emotional struggle their loved ones had been through during their cancer treatment away from home, but often couldn’t give specific examples of how their families are being supported psychologically	*‘My partner is here all the time, she understands the complexity of it all’* *‘She’s taking it hard, mentally and physically… she doesn’t really talk to many people at all, she keeps it to herself which is hard’* *‘My husband has been more stressed than I have… I’m his anchor I think… he won’t talk to anyone’*
*Yearning for rural life:* Missing home and rurality was raised several times, as was a desire to find a green space whilst away. For most, looking forward to having the weekend at home seemed to help improve their emotional well‐being. Some felt fortunate to be having treatment in a regional centre instead of a larger metropolitan centre	*‘When I come up here I miss home, it’s too far to go home every weekend and that’s the disadvantage I feel I’ve got with it… I do miss being away from home, I can’t do thing here like I do at home,* *‘There is no garden there… it’s a shame that there is no particular area, like a garden or open space where people can just go and meditate, look at the flowers and breathe the fresh air… Would be nice to meet others and have a little chat’*
*Social isolation:* One participant talked about increasing loneliness, social isolation and ageing in a small rural village. Another participant who had undergone five weeks of treatment alone, away from home, was relieved that he had someone to talk to during his interview	*‘I was at the younger age group of our group of friends and they’ve been gradually going… Those that haven’t died have gone to live with family, so it’s a bit lonely really. There is a whole different demographic around us, and they are people that I don’t know’* *‘It’s been good, get a bit off my mind’*
*Having little choice:* Some expressed anxiety about the feeling that they have little control over staying away from home for treatment. Others talked about the pressure of commitments they had a home To help reduce levels of anxiety, visualisation techniques or sound and light installations were suggested by a patient who struggled with the necessary immobilisation during RT	*‘I do sometimes (find it stressful), but I have to have this radiation because if they don’t stop it from growing then it’s going to kill me, simple as that. So there’s no choice, so I have to get over that’* *‘But my husband has PTSD… He has reached the stage where he doesn’t really like being around people at all, consequently I don’t leave him that much’* *‘Some of those (visualization) techniques would probably help a lot of people going though it, even if there was something there that you look at, you know like one of those surreal films where you look at an you actually feel like you are part of it… or whether music that you could sort of feel like you were swirling around, surround sound or whatever might help some people. Well it probably would have helped me’*
*Practical Supportive Care Needs:* Some stated it was practically impossible to stay away from home for the entire treatment because of work and caring commitments. Some participants grateful for nearby accommodation and adjusted appointment times. There was plenty of discussion about the practicalities of being away from normal services during RT	
Summary	*Interview Quotes*
*Appointments and transport:* Some experienced confusion and anxiety over appointments and would prefer routine times. One participant was distressed after missing an appointment because of a major accident Some participants mentioned trying to get jobs done or attend other appointments while they were in the metropolitan area. One had had their car serviced on the day of their CT appointment but delays prevented timely car collection and undue stress	*‘If they said this week is 11am (all week) then everything would be fine and you can sort of plan things a little better… you can work your life around the appointments’* *‘You can’t predict what the traffic is going to be like – it can take up to half and hour longer than what you expect’* *‘Couple of times the schedule for the nurses appointment is half a hour before the other one and then they just say the nurses will see you afterwards so you just sit there for an hour. But now I know to take no notice of the time’* *‘But you never know when you are driving to here (from home) because there is a huge great signal blackspot, so I probably could have gone a back road but I was worried that if I’d broken down there was no mobile reception and so I wouldn’t be able to call the hospital or home. So when you live so far away from a medical facility you have to take these things into consideration’*
*Knowing what to bring/what happens when you forget something:* As outsiders to the area, some patients would welcome practical local information. Knowing what to pack and remembering everything was a common conversation topic, with patients forgetting practical and other items, and even vital medications. Patients forgetting their medication had to pay ‘full whack’ for it nearby or travel home mid‐week. One patient went without his regular nicotine replacement therapy because of this	*‘I brought a book the first week but I realised I didn’t bring my reading glasses… I am diabetic so I take insulin – I bought two pens of each and I had picked up an old pen that I had already used it – so it was a quick drive home (110km)’* *‘It’s a bit of a hassle being away from my GP and pharmacy (my prescription was only valid 6 months not 12) and I ummed and aahed and I’ve gotta take a trip back to get a new prescription’* *‘We did (have to use a pharmacy locally) last week, which was a shame because we had to pay for it’* *‘They don’t tell you what you need to bring. You don’t find out until you get there. Probably took me a week to realise what I needed. I’ve got it figured out now that I’m finishing’* *‘It wasn’t entirely satisfactory because I normally have a Webster pack for me as well and they couldn’t do that because they didn’t have the right details’*
*Activities of daily living:* A daily routine away from home was important for many participants. This was not always practically possible or was hindered by symptoms and side‐effects. Some participants spent their time away from home exploring or visiting friends and family, which required access to a vehicle	*‘I don’t do my hobby here but I should have done… I didn’t bring my sewing machine the first time and I was going to bring it this time but we were running late. I would have been sewing, its not straining’* *‘Last week we visited a local town one day, then another the next day, this afternoon we’ll probably go for a drive somewhere… There’s always places to go. I want to take a look at another village but I don’t know how far away it is. I have been just asking the cleaning ladies’* *‘We’ve been down to town every day when my daughter has been (for one week) but I haven’t got a vehicle so I can’t go down shops in town I’d have to get a taxi’*
*Financial and technological concerns:* The financial strain of attending treatment away from home was raised multiple times. Eligible participants were grateful they could claim fuel vouchers but some were worried about correct form filling. Not all participants had regular access to the internet. Others had limited access to suitable telephones for Telehealth appointments while away from home or had to buy a new one	*‘The whole thing has been a bit of a drain, it will have cost us several thousand dollars even with the help, as we are still paying rent back home’* *‘I am on a tight budget, I just live to my means’* *‘I find it hard to keep records for buying fuel… If (home) was 90 kms away instead of 103 kms then we wouldn’t be able to get the vouchers, it’s crazy… we are pensioners… it is a lot of money to be paying out for fuel each week’* *‘I never had a (mobile) telephone. I had to buy a mobile… 3 weeks ago I got my first smart phone so I’m still trying to work out how to use it’* *‘My GP told me about the video conferencing appointments or something, but I don’t know anything about that. I had a computer at home, not hooked‐up to the internet, but it broke…’*
*Physical Supportive Care Needs:* During the interviews participants discussed their struggles to meet physical needs such as nutrition and sleep during RT. Their struggles were exacerbated by extended periods away from home. Where physical SCN were met, this has been because of regular nurse and doctor input providing clear understanding of what to expect	
Summary	*Interview Quotes*
*Diet, sleep, exercise and skin care*: Many participants faced fatigue, especially those with no family member or carer or who live alone as they have to do their own driving and domestic duties. Tiredness took its toll on those travelling long distances home each weekend. Practical matters such as accessing grocery shops can impact physical health causing fatigue, stress, and also encourages patients to purchase easy but unhealthy fast food.	*‘I’m tired after the drive home but I’ve got washing to do’* *‘I go home, I’ve got clothes to wash and the house to tidy up’* *‘The walking back, especially carrying groceries, is a bit tiring, because I haven’t been getting much sleep since I’ve been here… I’ve been running to the toilet much more often’*
*Co‐morbidities and pain:* One participant took strong painkillers and often overran her appointment time because she needed to rest her shoulder when she could not hold the position. This may have been exacerbated with only getting two‐day notice for her planning CT, arranging last‐minute accommodation, and a 9‐hour drive. She had not been advised to practise breath‐holding or lying down with her arms elevated and expressed regret about that Another participant described how the lack of specialist and regular GP in her local community had prevented her from getting timely medical help for a lung co‐morbidity. This impacted her comfort during RT	*‘The only hiccup has been the frozen shoulder….‘There had been a mix up… Possibly we’d have done it different if we’d have known – I don’t know’* *‘The last time I was at the (lung) specialist’s clinic he said he would have referred me to a respiratory physio but the one here had just been promoted and so no longer does it here, but (now I’m finished RT) I am going to talk to my doctor when I see her again, and ask her to see in there is a different respiratory physio somewhere else… My GP keeps changing’*

## Discussion

This study set out to explore patient experiences being away from home in order to better understand them, thus giving health care professionals a deeper appreciation of how to best meet their SCN. Each participant’s experience is recognised as unique and fluid, influenced by time, space, ideals and relationships.^20^

The evidence here suggests that a more thorough understanding of patient experiences can be gained with consideration of their current values and identities. It is important to recognise that these can change as undergoing diagnosis and treatment has been shown to impact personal identity.^21^ For those who place a high value on health care, combined with staying far away from their home, ‘being a patient’ can actually become an integral part of their identity.^22^ The interviews here were not designed to explore this in depth, and further exploration is suggested since existing research on patient identity is largely focussed on survivorship.

Understanding a patient’s values reveals their priorities during treatment. Values may not be explicitly stated but, with time and trust, a story told may provide insight into identity. Health care professionals will then be better equipped to meet patient individual needs. Giving patients space to tell their story improves well‐being,^23^ and staff should be proficient active listeners and know how to make appropriate referrals to supportive care services.

The experience of attending RT away from home was often associated with health care fatigue, severe exhaustion, confusion and stress, but some patients expressed a sense of relief that they were finally in the right hands and having active treatment. This supports a growing body of evidence demonstrating that rural individuals accessing cancer care experience their SCN in a complex and dynamic way.^9^

The resilience of participants has been supported previously for rural patient populations.^24^ A resilient attitude has been linked to a more stoic nature, suggesting patients are less likely to seek and accept support.^25^ This is not always the case, and patients might not consider themselves to be resilient by definition.^26^ Even when an individual has overcome devastating life events, they may still fear being away from home for treatment, suffer from health care fatigue or loss of control. This study shows this is especially the case if expectations are not well‐managed.

Effective patient‐centred care means working with them to better meet their individual needs adapting their ongoing SCN. Clear communication between all parties is key. Prior studies suggest embedding a supportive care framework into departmental policy.^27^ Patients expressed a sense of comfort in having a regular GP, pharmacist and team of specialists providing efficient and clear communication. Previous research^28^ notes that rural cancer patients benefit greatly when there is effective collaboration and co‐operation between inter‐disciplinary teams. It highlighted the decline in support following the completion of intensive treatment. Rural patients may need more support for effective self‐monitoring practices than those living nearby. Effective self‐monitoring should be encouraged and supported consistently by health care teams, and it is recommended that consideration is given to a rural patient’s cognitive, affective, interpersonal and symptomatic factors.^29^

In health care, social support cannot necessarily be separated from psychological support.^30^ Experiences of valuable psycho‐social support have not always been formally noted; supportive care can be delivered through positive social interactions by anyone at the facility including administrative staff, cleaners and other patients/visitors. Patients experiencing caring professionals who took their time to get to know them seemed happier and safer away from home. This is supported by established work developed by Eckermann et al.^31^ promoting culturally safe environments in Aboriginal Health. This environment is characterised by active listening, shared meaning, respect, shared knowledge and experiences, with a focus on learning together with dignity. Recent research on indigenous health staff of two high‐performing oncology departments demonstrated that improved patient outcomes can be achieved through a strong culture of respect and two‐way learning.^32^ Their holistic evaluation demonstrated that cultural safety for patients, carers and staff depends heavily on clear leadership.

Having supportive family/friends was comforting for most participants. Patients often perceived that their loved ones were shocked by the diagnosis, anxious and distressed as they undertook treatment away from home. There appears to be a lack of support for patient families during this difficult time.^6^ A study designed to explore the caregiver’s perspective suggested that support should be tailored and culturally appropriate, especially in regard to privacy preferences.^33^

The experience of treatment away from home can be daunting, lonely and frightening. During treatment, patients may lose part of their identity. Continuing normal activity, a hobby or something familiar helps maintain a sense of home. Previous research on breast cancer patients indicates that psycho‐social support can be gained from hobbies/habits onwards into survivorship.^34^

Cancer treatment away from home is never convenient, but having easy access to practical solutions appears to provide many patients with a sense of calm and reduced anxiety during such a challenging time. Being able to go home at weekends, having access to a vehicle and having someone to visit them were important points raised by participants. This has been indicated by prior research, and patients should be practically supported.^35^ Yet, this study has demonstrated that some patients prefer to be alone, perhaps to have time to focus on themselves and their treatment and thus experience a sense of control over their own SCN with reduced anxiety. Using effective communication to manage expectations seems to benefit cancer patients’ feelings of control.^36^ Furthermore, rural patients who practise self‐monitoring, guided by nurses, foster a greater sense of control and self‐advocacy,^30^ features which were also valued by patients interviewed here.

It has been previously documented that cancer patients have significant levels of unmet health‐system informational needs.^37^ Here, some of the rural patients who stay away from home talked about negative experiences of mistimed and confusing information. Despite a preference for face‐to‐face communication, this was less likely because of travel distances and technological constraints. Recent work has been done to manage expectations through a series of online videos capturing rural patient experiences of cancer.^38^ Participatory action research also suggested that sharing rural patient stories may encourage others to be more open and help manage expectations.^39^ However, there remain major educational, practical and technological constraints for this patient population, including restricted access to wifi, smartphones or computers. Conversely, becoming adept at accessing relevant information via online platforms is not a priority for some.

This study revealed what patients would recommend to others, for example, treatment‐specific information on how to practise DIBH at home, or maps of the local area. Ugalde et al.^28^ found that rural carers also require practical support navigating the health system, arranging accommodation and travel to help maintain their own well‐being and focus on supporting the patient. Practical evidence‐based supportive care for both patients and carers is paramount.

### Recommendations

Patient perspectives were demonstrated by five interlaced themes; all could warrant much further research. The key recommendations are outlined in Table 6, some of which could apply to all cancer patients and those who stay away from home. The issues were identified by exploring real patients’ experience. It is vital that future study is focussed on better supporting their practical, psychological, social or physical needs.

**Table 6:** Summary of key recommendations.

	Recommendations for action
1	Health care professionals should be given the resources (time and training) to effectively engage with patients who spend time away from home about their individual values and beliefs during treatment
2	Patient notes should start with a clear summary, medical history and up‐to‐date social history to give health professionals a clearer picture of the supportive care needs (SCN) of these patients
3	Regular and open inter‐disciplinary communication, meetings and training to reduce the chances of misinterpreting a patient’s SCN as they spend time away from home
4	Regular contact with the same health care team would aid the patient to build trust and communicate better about whether their needs are being met during treatment away from home
5	Provide tailored practical support to ensure patients are supported socially during treatment away from home
6	Issue a packing checklist for radiation therapy treatment away from home
7	Give an overview of the local area, support groups and other resources which could encourage the continuation of a hobby or interest
8	Give clear informational support fitted to the individual (preference for written or verbal, and access to technology). This will help manage expectations during time away, encourage patient engagement and provide a sense of control over their own treatment (e.g. the DIBH technique)

	Recommendations for the focus of future research
1	Further study of how a patient’s identity and values change as they go through treatment away from home
2	Focus the research more on specifically how best to manage expectations for rural patients who have to spend significant periods of time away from home
3	Further research on patient perceptions of their own SCN at different times to give a longitudinal perspective, exploring the patient experiences of returning home after RT and their self‐monitoring practices over time
4	Explore the unmet SCN of carers during diagnosis and treatment
5	Further study of the impact of promoting cultural safety through clear leadership on the SCN of vulnerable rural/remote patient populations

### Limitations

The constant comparative method of data analysis allowed the researcher to identify when information saturation occurred, that is, additional interviews would not have added to the richness or quality of the data. This can be difficult to pinpoint, especially with a novice researcher. An action plan was followed, and the rigorous method was made as transparent as possible. Credibility was provided by using the analysis process outlined by the literature.^17^, ^19^ Input was sought from the novice researcher’s colleagues during the process to aid data collection, reflexivity, analysis and evaluation. The research worked within the limits of the resources available; nevertheless, it would have benefitted from having multiple experienced researchers or a secondary interviewer/coder to triangulate the data.^18^

## Conclusion

This study explored the lived experiences of patients as they attend treatment away from home. Patients discussed their own SCN, and three themes emerged: practical, physical and psycho‐social. Patients indicated how their values, identities and expectations have influenced their experiences. The complexity of SCN should not be underestimated, especially for those vulnerable to the negative effects of social isolation. Yet, it has been demonstrated that patient well‐being is hugely influenced by the compassionate, caring and respectful connections they make during treatment. Future studies must work towards efficient and holistic patient‐centred care, which is culturally safe for all parties involved.

## Conflict of Interest

No conflict of interest has been declared by the authors.

## Ethics Approval Statement

This research has been approved by the North Coast NSW Human Research Ethics Committee.

## Patient Consent Statement

Written consent was obtained from all patients who participated in this research. Personal identifiers were removed at the time of transcribing the interviews.

## Funding Information

This research received no specific grant from any funding agency in the public, commercial or not‐for‐profit sectors.

## Supporting information


**Appendix S1.** Literature review.

**Appendix S2.** Evaluation of methodology.

**Appendix S3.** Interview guide.

## Data Availability

The data that support the findings of this study are available from the corresponding author upon reasonable request.

## References

[1] Harrison J , Young J , Price M , Butow P , Solomon M . What are the unmet supportive care needs of people with cancer? A systematic review. Support Care Cancer 2009; 17: 1117–28. 19319577 10.1007/s00520-009-0615-5

[2] McElduff P , Boyes A , Zucca A , Girgis A . Supportive Care Needs Survey: A Guide to Administration, Scoring and Analysis. Newcastle, Australia: Centre for Health Research & Psychology (CHeRP), 2004.

[3] Hack T , Pickles T , Ruether J , et al. Predictors of distress and quality of life in patients undergoing cancer therapy: impact of treatment type and decisional role. Psychooncology 2010; 19: 606–16. 19557823 10.1002/pon.1590

[4] Fitch MI , Maamoun J . Unmet supportive care needs and desire for assistance in patients receiving radiation treatment: implications for oncology nursing. Can Oncol Nurs J 2016; 26: 53–9. 31148742 10.5737/236880762615359 PMC6516328

[5] Soanes L , Gibson F . Protecting an adult identity: a grounded theory of supportive care for young adults recently diagnosed with cancer. Int J Nurs Stud 2018; 81: 40–8. 29455009 10.1016/j.ijnurstu.2018.01.010

[6] Clavarino AM , Lowe JB , Carmont SA , Balanda K . The needs of cancer patients and their families from rural and remote areas of Queensland. Aust J Rural Health 2002; 10: 188–95. 12121408 10.1046/j.1440-1584.2002.00436.x

[7] Fitch MI , Gray RE , McGowan T , et al. Traveling for radiation cancer treatment: patient perspectives. Psychooncology 2003; 12: 664–74. 14502591 10.1002/pon.682

[8] Lisy K , Langdon L , Piper A , Jefford M . Identifying the most prevalent unmet needs of cancer survivors in Australia: a systematic review. Asia Pac J Clin Oncol 2019; 15: e68–78. 31215167 10.1111/ajco.13176

[9] Loughery J , Woodgate RI . Supportive care needs of rural individuals living with cancer: a literature review. Can Oncol Nurs J 2015; 25: 2368–8076. 10.5737/23688076252157166 26638285

[10] Australian Government Department of Health . Modified Monash Model ‐ fact sheet. [cited 2021 June 20]. https://www.health.gov.au/resources/publications/modified‐monash‐model‐fact‐sheet

[11] Grimison P , Phillips F , Butow P , et al. Are visiting oncologists enough? A qualitative study of the needs of Australian rural and regional cancer patients, carers and health professionals. Asia Pac J Clin Oncol 2013; 9: 226–38. 22989350 10.1111/ajco.12014

[12] Sabesan S , Brennan B . Tele oncology for cancer care. In: Graschew G , Rakowsky S (eds). Rural Australia, Telemedicine Techniques and Applications [e‐book]. IntechOpen, 2011 [cited 2019 Mar 15]. Available from: https://www.intechopen.com/books/telemedicine‐techniques‐and‐applications/tele‐oncology‐for‐cancer‐care‐in‐rural‐australia

[13] RTOC Radiation Oncology Tripartite Committee . Supporting regional and rural access to radiation oncology services: planning for the best: tripartite national strategic plan for radiation oncology 2012–2022. 2012 [cited 2020 Mar 12]. Available from: http://www.radiationoncology.com.au/rural‐and‐regional‐access/

[14] NSW Government . Partners in health: a community engagement framework for northern NSW local health district. 2019 [cited 2019 Mar 03]. Available from: https://nnswlhd.health.nsw.gov.au/wp‐content/uploads/2019_Community‐Engagement‐Framework_online_FINAL‐1.pdf

[15] Saunders B , Sim J , Kingstone T , et al. Saturation in qualitative research: exploring its conceptualization and operationalization. Qual Quant 2018; 52: 1893–907. 29937585 10.1007/s11135-017-0574-8 PMC5993836

[16] Ballinger B . Demonstrating rigour and quality. In: Finlay L (ed). Qualitative Research for Health Professionals: Challenging Choices. John Wiley & Sons, Chichester, UK, 2006; 235–46.

[17] Alase A . The interpretative phenomenological analysis (IPA): a guide to a good qualitative research approach. Lit Numer Stud 2017; 5: 9–19.

[18] Tong A , Sainsbury P , Craig J . Consolidated criteria for reporting qualitative research (COREQ): a 32‐item checklist for interviews and focus groups. Int J Qual Health Care 2001; 19: 349–57. 10.1093/intqhc/mzm042 17872937

[19] Lincoln YS , Guba EG . Naturalistic inquiry. Beverly Hills: Sage, 1985.

[20] Benner P . The tradition and skill of interpretive phenomenology in studying health, illness, and caring practices. In: Benner P (ed). Interpretive Phenomenology: Embodiment, Caring, and Ethics in Health and Illness. Sage, London, UK, 1994; 99–127.

[21] Haslam SA , Jetten J , Postmes T , Haslam C . Social identity, health and well‐being: an emerging agenda for applied psychology. Int J Appl Psychol 2009; 58: 1–23.

[22] Thong M , Wolschon EM , Koch‐Gallenkamp L , et al. "Still a cancer patient"‐associations of cancer identity with patient‐reported outcomes and health care use among cancer survivors. JNCI Cancer Spectr 2018; 2: 31. 10.1093/jncics/pky031 PMC6649846 31360857

[23] Brown G , de Jong J . Cancer storytelling: a study of well‐being expressions made by patients. J Pastoral Care Counsel 2018; 72: 37–44. 29623806 10.1177/1542305018754796

[24] Pham TV , Beasley CM , Gagliardi JP , Koenig HG , Stanifer JW . Spirituality, coping, and resilience among rural residents living with chronic kidney disease. J Relig Health 2020; 59: 2951–68. 31392626 10.1007/s10943-019-00892-w

[25] Butow PN , Phillips F , Schweder J , et al. Psychosocial well‐being and supportive care needs of cancer patients living in urban and rural/regional areas: a systematic review. Support Care Cancer 2012; 20: 1–22. 10.1007/s00520-011-1270-1 21956760

[26] Tan WS , Beatty L , Koczwara B . Do cancer patients use the term resilience? A systematic review of qualitative studies. Support Care Cancer 2019; 27: 43–56. 30209600 10.1007/s00520-018-4456-y

[27] Fitch MI . Supportive care framework. Can Oncol Nurs J 2008; 18: 6–24. 18512565 10.5737/1181912x181614

[28] Haigh MM , Baxi S , Lyford M , Cheetham S , Thompson SC . Cancer support services: Are they meeting the needs of rural radiotherapy patients? Eur J Cancer Care (Engl) 2019; 28: e12904. 30084525 10.1111/ecc.12904

[29] Purtzer MA , Hermansen‐Kobulnicky CJ . 'Being a Part of Treatment': The meaning of self‐monitoring for rural cancer patients. Cancer Nurs 2013; 36: 93–103. 22964865 10.1097/NCC.0b013e318263f385

[30] Legg MJ . What is psychosocial care and how can nurses better provide it to adult oncology patients. Aust J Adv Nurs 2011; 28: 61–7.

[31] Eckermann A , Dowd T , Chong E , Nixon L , Gray R , Johnson S . Binang Goonj: Bridging Cultures in Aboriginal Health, 3rd edn. Elsevier, Sydney, NSW, 2010.

[32] Taylor EV , Lyford M , Parsons L , et al. “We’re very much part of the team here”: a culture of respect for Indigenous health workforce transforms Indigenous health care. PLoS One 2020; 15: e0239207. 32960933 10.1371/journal.pone.0239207 PMC7508383

[33] Ugalde A , Blaschke S , Boltong A , et al. (2019) Understanding rural caregivers’ experiences of cancer care when accessing metropolitan cancer services: a qualitative study. BMJ Open 2019; 9: e028315. 10.1136/bmjopen-2018-028315 PMC6629412 31300501

[34] Tominaga K , Andow J , Koyama Y , et al. Family environment, hobbies and habits as psycho‐ social predictors of survival for surgically treated patients with breast cancer. Jpn J Clin Oncol 1998; 28: 36–41. 9491140 10.1093/jjco/28.1.36

[35] Vindrola‐Padros C , Brage E , Chambers P . On the road and away from home: a systematic review of the travel experiences of cancer patients and their families. Support Care Cancer 2018; 26: 2973–82. 29796709 10.1007/s00520-018-4266-2

[36] Zachariae R , Pedersen CG , Jensen AB , et al. Association of perceived physician communication style with patient satisfaction, distress, cancer‐related self‐efficacy, and perceived control over the disease. Br J Cancer 2003; 88: 658–65. 12618870 10.1038/sj.bjc.6600798 PMC2376357

[37] Sanson‐Fisher R , Girgis A , Boyes A . The unmet supportive care needs of patients with cancer. Cancer 2000; 88: 225–36. 10.1002/(sici)1097-0142(20000101)88:1<226::aid-cncr30>3.3.co;2-g 10618627

[38] University of South Australia . Media release. 2018 [cited 2020 Jan 12]. Available from: https://www.unisa.edu.au/Media‐Centre/Releases/2018/Cancer‐stories‐a‐lifeline‐for‐rural‐cancer‐patients‐and‐survivors/

[39] Gunn . The psychosocial needs of rural cancer patients. 2013 [cited 2019 Mar 8]. Available from: https://digital.library.adelaide.edu.au/dspace/bitstream/2440/81928/8/02whole.pdf

