# Supplementary material for: Being away from home for cancer treatment: a qualitative study of patient experience and supportive care needs during radiation therapy
Source: J Med Radiat Sci. 2022 Apr 4;69(3):336–47. doi: 10.1002/jmrs.578 (PMC9442298; doi:10.1002/jmrs.578)
Supplement: Supplementary file 1 — Appendix S1. Literature review. [file JMRS-69-336-s003.pdf]

Supporting Information 1 - Literature Review

Being away from home for cancer treatment: A qualitative study of patient experience and supportive care needs during radiation therapy

Authors: Vanessa Knibbs 1, BScPsycMgt, PostGradDipRad; Stephen Manley 1, MBusAdmin, BApplSci-MedRad, DipProjMgt, GradCertBus
Affiliations: 1 North Coast Cancer Institute, Lismore, Northern NSW LHD. Corresponding Author Address: ness_knibbs@yahoo.co.uk

Needs of Cancer Patients

Review of 2 systematic reviews and a large scale study from NSW

1

Background

Unmet needs defined as requirement of some action or resource that is necessary, desirable or useful to attain optimal well-being

QoL research shows diagnosis and treatment of cancer impairs patient's:

- work and social activities
- management of the home
- family and other relationships
- sleep patterns
- sexual activity
- levels of anxiety and depression

2

Measured Patient Satisfaction

- High for clinical aspects of care
- Low for information and support about:
  - disease
  - treatment
  - side effects/side effect control
  - support at home

3

The unmet supportive care needs of patients with cancer *

- Conducted by the NSW Cancer Council supportive care review group and the Cancer Education Research Program
- Aim: Describe the prevalence of unmet needs among a large sample of cancer patients undergoing different types of treatment for their cancer at different treatment centers
- Disease and treatment variables were examined as predictors of different types of unmet needs

* Sanson-Fisher, R, & Girgis, A, & Boyes, A. (2000) The unmet supportive care needs of patients with cancer. *Cancer*. 88(1):225-236.

4

Sample

- 9 treatment centres (55% of all NSW RT centres, 3 of which offered rural RT clinics)
- Patients diagnosed at least 3 months prior
- 18-85years, English reading/writing and speaking
- Supportive care needs survey and reply paid envelope and trained interviewer to explain what it was for
- Modified version of the Cancer needs questionnaire (pilot tested in a prior study using 200 subjects) 71 questions in 3 groups:
  - 5 separate factors associated with need
  - Disease and treatment
  - Patient background

5

Results - 888 surveys

- Highest levels of unmet need found in the psychologic domain, then health system and information and then physical and daily living domain.
- Patients in remission had fewer needs across all domain than those not in remission.
- Females reported higher levels of unmet need in the psychologic and care and support domains than males.
- Treatment centre was identified as a significant predictor of reporting some unmet needs.
- Patients with multiple sites, lung, colon, rectal or brain cancer had higher needs than breast.
- Time since last admission predicted level of unmet physical needs.

6

Discussion

- One similar prior study had found information to be the most common unmet need, suggesting this might have improved over time.
- Psychological need has been identified before by numerous other studies.
- Discussed the range of interventions aimed at improving psychosocial support such as tailored counselling delivered by specialist oncology health professional or relaxation training for patients undergoing radiation therapy.
- It is likely that the needs of some cancer patients will never be fully met and a level of perceived unmet need will always be present.

7

Recommendations

- Develop interventions which look at:
  - Structural changes to the provision of care
  - Improving the interactional skills of health professionals
  - Improving the provision of or access to resources
  - Providing feedback to clinicians
- Clear and regular monitoring to ensure oncology care can best meet the needs of a variety of patients.

8

Summary

- Does take into account the context of rural/urban centres.
- The patients were only considered at one time point in their diagnosis, which was not the same for all patients.
- 'Some need' was categorised as those patients reporting a 'moderate' or 'high' unmet need and only the top ten ranking needs were reported, what about the other needs?
- In comparison to the population the 888 sample overrepresented females; cases of breast, bowel, colon and rectal cancer; and those ages 31-60 years.
- Underrepresented males; prostate carcinoma, lung carcinoma, and skin cancer/melanoma cases; and patients ages 71-85 years.
- 66% response rate - low

9

What are the unmet supportive care needs of people with cancer? Systematic review *

- Supportive care can be defined as care that helps a person with cancer and their family cope with cancer and its treatment, from pre-diagnosis through the process of diagnosis and treatment to cure, continuing illness or death and into bereavement. Aims:

1. ascertain the prevalence of unmet supportive care needs in adult cancer patients according to time point of the cancer illness;
2. investigate differences in unmet need for different tumour groups and different stages of disease;
3. identify clinical and personal predictors of unmet need;
4. document study design investigating prevalence of unmet need

10

The most frequently reported unmet SCN

- Activities of daily living domain (1-73%)
- Psychological information (6-93%)
- Psychosocial (1-89%)
- Physical (7-89%)

- 57 quantitative studies
- Excluded kids/young adults, carers, non-English speakers
- Satisfaction studies and Studies translated into English (non-Australian studies) were included
- QoL studies were not included

* Harrison, J.D. et al (2009) What are the unmet supportive care needs of people with cancer? A systematic review. *Support Care Cancer*. 17:1117-1128.

11

SCN while on Treatment

- The highest levels of unmet need for most domains were identified during treatment
- The prevalence of unmet need for each domain had the largest variation during the treatment phase compared to any other time point of the cancer illness
- Predictors unmet needs: low social support networks, low income, increasing age, decreasing age (<60 years), advanced disease and not being told that the cancer was diminishing, being geographically isolated from health services

12

Summary

- Some need definition is not universal
  - One factor may be ticked and some need is present
- Measures are hardly ever taken across time periods
- The results could be skewed because there were more studies made during treatment
- Call for more uniform measuring of unmet need

13

Supportive care needs of rural individuals living with cancer: A literature review *

- Large scale study of 23 international papers both qualitative and quantitative
- Studies frequently reported that the cancer experience creates significant psychological and emotional disruptions for patients and their families including:
  - Lack of services rurally
  - Difficulties with practical needs
  - Uncertainty and fear around travelling
  - Lack of privacy leading to isolation
- 7 studies identified negative impact of social isolation
- A need to discuss emotional concerns with people in similar situations was commonly identified

14

Positive influence

- This literature review also highlighted the numerous positive features of rural life patients have highlighted:
  - positive culture and supportive community networks
  - sense of peace
  - learned survivorship
- Positive aspects of staying in comfortable, social and affordable hospital accommodation similar to Our House

* Loughery, J & Woodgate, R.I. (2015) Supportive care needs of rural individuals living with cancer: A literature review. Canadian Oncology Nursing Journal. 25(2): 2368-8076.

15

Summary

- Research demonstrates high prevalence of unmet needs.
- Good social support is often related to fewer unmet needs.
- The literature suggests that people with more advanced stages of disease or with poor health status are more likely to have unmet needs.
- Lung cancer was also identified as a predictor of higher levels of unmet need.

16

Further Questions

- Are rurally based patients at a higher risk of having unmet supportive care needs?
- Are we measuring the unmet needs of our patients across all domains?
- How we can assess their level of need across the patient's pathway?
- How can we address those unmet needs best? **Ask the patients/carers**

17
