# Supplementary material for: Being away from home for cancer treatment: a qualitative study of patient experience and supportive care needs during radiation therapy
Source: J Med Radiat Sci. 2022 Apr 4;69(3):336–47. doi: 10.1002/jmrs.578 (PMC9442298; doi:10.1002/jmrs.578)
Supplement: Supplementary file 2 — Appendix S2. Evaluation of methodology. [file JMRS-69-336-s002.docx]

**Supporting Information 2 - Evaluation of Methodology**

**Being away from home for cancer treatment: A qualitative study of patient experience and supportive care needs during radiation therapy**

Authors: Vanessa Knibbs ^1^, BScPsycMgt, PostGradDipRad; Stephen Manley ^1^, MBusAdmin, BApplSci-MedRad, DipProjMgt, GradCertBus

Affiliations: ^1^ North Coast Cancer Institute, Lismore, Northern NSW LHD.

Corresponding Author Address: [ness_knibbs@yahoo.co.uk](mailto:ness_knibbs@yahoo.co.uk)

*Research Paradigm*

The research paradigm framed the question and informed the design of the research in a logical manner. An interpretive phenomenological approach was taken, which means the research was not about uncovering one truth, but was trying to understand the essence of the phenomenon by examining the views of those experiencing it. Patterns were examined across multiple participants to understand the nature of the experience. The researcher aimed to shed light on the subject by making interpretations and constructions of the patient perspectives during the phenomenon of interest (attending for lengthy cancer treatment away from home). Inductive and exploratory in nature, there was a constant comparative method of data analysis. The emergent themes help characterise a narrative of experience, transitioning the data from abstract to conceptual.

The chosen approach has been influenced by the researcher’s own experience, values and beliefs; that there are multiple socially-constructed realities which are experienced differently by different people and influenced by context. So while an interpretivist approach was taken it was also heavily influenced by social constructivist ideals. The different approaches that could have been taken include interpretivist, constructivist, positivist and critical theory. Yet it is important to remember that these are not incompatible or mutually exclusive (1). Table 1 gives a brief overview of these main paradigms, and how they might reframe this research.

| Paradigm | Question | Critique |
| --- | --- | --- |
| Positivist: realist in nature; assume that there is a stable reality separate to the researcher’s understanding of it and seek an observable, rational and objective reality (2). | What type of SCN do rural patients who stay away from home for cancer treatment have? | SCN research grounded in positivism has been criticised for the exemption of contextual information such as spiritual, sexual, and financial concerns (3). Taking a positivist approach would risk over-simplification of the phenomenon. |
| Social constructivist: emphasis on how the phenomenon has been constructed as a result of significant historical, cultural, social or political processes. | How do patients who stay away from home interpret the medical construct of SCN? | Whilst it is important to recognise the constructs within health care cannot be removed from the social world it appears within, it must not become restricted by these either (4). |
| Critical-theory: places the social phenomenon fully within the historical context, its ideologies and processes. Requires a careful and critical understanding of contextual factors. | How does rural community stoicism influence the perspectives of SCN patients who stay away from home for cancer treatment? | By placing the sole focus on a social or historical phenomenon of interest such as rural stoicism (5), alternate perspectives may be missed. This approach may require a considerable shift of focus for the lead-researcher, who has little first-hand experience of living in a rural or remote area of Australia. |

**Table 1:** Alternative research approaches

*Methods*

The published literature on the topic of SCN for cancer patients indicates a complex issue suitable for in-depth qualitative inquiry, which is reinforced by the research paradigm. Interviews are the most common qualitative method of exploring the SCN of rural cancer patients (6) and appear to be widely accepted in health research (7). Interviews can be structured, semi-structured or unstructured. Semi-structured interviews allow the exploration of the phenomenon in great detail; the aim is to encourage conversation, guided by the patient’s emerging viewpoints. An interview guide (Supporting Information 3) was developed to ensure commonly measured topics of SCN are discussed for all participants (8). Brown Lui, Robinson, & Boyle (9) also used these domains to guide their interviews with lung-cancer patients; unfortunately other qualitative studies do not provide detail on how their interviews were directed. This guide has been reviewed by a number of appropriate health professionals to provide professional validity. Despite a guide being used, the interviews were not semi-structured - a conversational approach was used to ensure patients were comfortable to openly discuss their own SCN perspectives.

Semi-structured interviews with lengthy discussions of relevant topics resulted in large amounts of raw data, which lead to more labour-intensive analyses than structured interview data. However, by taking a phenomenological approach, and judging by the lack of prior qualitative research on this cohort, it was important not to impose such methodological boundaries. Interviews were conducted in a safe environment, suitable for the flow of conversation (2). The environment was consistent for each interview.

*Sample*

Purposeful typical-case sampling means the researcher and the research supervisor used their prior knowledge about the purpose of the study so that they could approach suitable participants. The sample was expected to illustrate themes generated from real-patient perspectives during treatment (2) thus the research can reasonably apply it’s conclusions to other groups of similar patient populations.

Sampling continued until information redundancy or saturation occurred and a timeframe and action plan was outlined to avoid premature saturation (10). Data collection and analysis occurred simultaneously so that the researchers knew when this point was reached. It was decided that if this point could not be easily recognised, sampling would continue until 15 interviews were completed (based on an average sample-size of 65 peer-reviewed qualitative research that used the same data analysis method (11)).

*Alternative Methods*

Qualitative exploratory research can be achieved in numerous different ways, an exhaustive review of which is beyond the scope of this discussion. Some SCN literature used focus groups; Wilkes et al. (12) conducted focus groups to explore the practical needs of rural cancer patients and carers attending treatment in a metropolitan centre. The focus groups highlighted a number of problems that were missed by surveys and practical solutions were able to be posed by the patients and families themselves, such as community-led informational support. However patients may not be willing to discuss less practical but more emotive SCN with a group of unfamiliar people. Focus groups can overly rely on good group dynamics and the NCCI does not currently have links with established groups of this particular cohort.

Duggleby et al. (13) used both interviews and focus groups to study the transitional experience of older rural patients with advanced cancer. Unstructured interviews were performed with patients and with family members; focus groups were conducted with health-care professionals. It highlights the idea that different types of participants may suit different types of data collection methods. This research project does not discount the opinions of others but it is bound within the limits of an achievable scope. Future research may consider the perspectives of others, such as carers or social workers, using appropriate methods such as focus groups.

Interviews and focus groups both rely on the participant’s ability to understand the questions/topics and effectively articulate themselves in English. Measures were taken to ensure meanings were understood accurately (active listening, checking for understanding and appropriate use of setting and notation of non-verbal cues). However, it could be argued that additional methods such as photo journaling would result in a more holistic exploration of the patient perspectives of being away from home. Edwards and Greeff (14) used photo-voice methodology to allow colloquial narratives to be documented in their study of cancer challenges faced by patients and families across South Africa involving 316 participants from 105 different communities. Here the study is limited to a location where <1% of residents reported poor proficiency in English (15).

Despite the fact that the research question does not fit well with quantitative positivist approaches, it could be enhanced further through the use of mixed methodology in a similar way to Clarvarino et al. (16), whose study of the needs of rural cancer patients and their families conducted in-depth qualitative interviews teamed with the SCN Survey. Mixed methods could have been used to ensure meanings assigned to perspectives are cross-checked, supported, enhanced, and ultimately validated.

Repeat interviews are useful in studying the transition of a phenomenon (13) or in longitudinal studies where perspectives are compared before, during and after travelling for treatment (17). Although it must be recognised that this dimension of Fitch et al.’s (17) research findings came from sampling across the 12 months after the introduction of a re-referral programme, thus its longitudinal data was generated in a non-deliberate convenient way.

**Data Analysis**

The data was subject to interpretive phenomenological analysis (IPA), described by Ricouer in 1976 (18) using three parts: naive understanding; structural analysis; and comprehensive understanding and reflection. Reflexivity gave the interpretation process credibility and transparency (19). Memos were made noting initial interpretations and insights; the same researcher built a set of categories representing potential points of interest and has sorted the data according to these. A thorough review then searched for higher levels of meaning, patterns/themes. This rigorous but time-consuming process involved adding categories and seeking more data until additional data didn’t meaningfully further contribute.

A clear example of IPA is demonstrated by Jumissko, Lexell, and Söderberg (20), who explored the meaning of family members’ experiences of living with those having suffered traumatic brain injury. It recognises the importance of considering their narrative as one interpretation of the experience; the true lived-experience cannot be transferred to the researcher completely. With IPA the demographics of the participants should not be a focus of the findings. The important characteristics of the participants include their exposure to the phenomenon to be studied and their ability to talk about it.

A recent use of IPA on more than 30 telephone interviews with cancer patients suggested that effective person-centred care is reliant on a deeper understanding of their individual needs (21). It performed an interesting secondary analysis which allows for a rigorous examination for alternative perspectives. Questionably, one of the aims of the study was to find out if people with cancer talk about the fundamentals of care, however the researchers only selected interviews with rich data discussing the fundamentals of care, thus already skewing the picture. Furthermore, with secondary analysis it must be acknowledged that the interview questions were not designed with the purpose of the study in mind. The data generated here could be subjected to secondary analysis in future depending on research need, ethical support and resource availability.

Interpretive content analysis would have much less focus on the lived-experiences of patient SCN away from home. Content analysis may be more suitable for understanding informational/practical needs and data from outside sources such as websites (22). IPA is different to discourse analysis, where the focus is on the function of the words used in a particular context (11). Grounded theory analysis offers a similarly rigorous process to IPA, but the construction of a theory explaining the phenomenon is the main goal (2; 23). This theory is then used to generate and test hypotheses. It will not be used here due to the limited resources and scope. Alternatively a top-down analytical approach could have been chosen, whereby existing theory is used as a starting point to categorise the data.

Nevertheless, IPA is a proven technique popular in healthcare research as it aids the understanding of complex social and psychological processes (11). It complements the research question, approach and the researcher’s values.

**Methodological Consistency**

The criteria adopted to demonstrate qualitative research validity and reliability must be compatible with the research question and the approach. The IPA guide published by the Australian International Academic Centre (24) was used to ensure a sound and thorough approach was taken. Analysis of the methods used were guided by a framework identified by Lincoln & Guba (25) and more recently by Ballinger (26) in her guide to qualitative research for allied health professionals. Furthermore a full checklist (27) for explicitly and comprehensively reporting this qualitative study can be found in appendix 2.

A preliminary study was conducted with 5 patients. The purpose of this was to assess the efficacy of the research methodology and prepare the researcher with the necessary tools and experience to ensure high quality study data is collected (24). The data collected during the pilot was recorded, the interview questions and research methods remained largely unchanged as a result of the pilot, therefore the data was incorporated into the main research study.

Brown et al. (9) were able to provide methodological rigour with independent coding by two researchers; disagreements were able to be discussed. However, they give no detail on how researcher bias was managed and whether the characteristics, values and beliefs of the researchers were even considered.

The process of bracketing undertook here is summarised in figure 1. Reflexive dialogue with colleagues was performed and a journal recording emerging personal/professional biases and previous knowledge was kept (28, see appendix 1 for an example). The researcher considered bias while interpreting perceptions of the participants and the phenomenon of interest was then challenged on its own terms (29). This is important for bottom-up IPA and where there is little prior theoretical base to demonstrate transparency and improve consistency (19).

Figure 1: Process of bracketing (30, 31)

Separating the phenomenon from the world and examining it closely

Reflexivity

Unravelling the structure by defining it and analysing it thoroughly

Suspending all preconceptions regarding the phenomenon

**Professional, Ethical, Practical factors**

The scale and scope of the project was agreed with the researcher’s manager. Methodology was influenced by the resources allocated; increased methodological quality was requested (secondary independent IPA, the inclusion of carers in the data collection) but deemed beyond the scope of the resources currently available.

The research has been limited to patients undergoing RT not just because it is those patients who often stay away from home for weeks and are considered vulnerable to unmet SCN, but also because the researcher is a radiation therapist and has close professional contact with these patients. Care has been taken to minimise the risk of a therapeutic misconception. The safe and effective care of the patient always takes priority.

Protecting participants from harm and upholding their human rights are major ethical concerns for any research. The research ensured all participants understood the study, its requirements and the risks involved before they consented to take part. A participant information sheet clearly stated the purpose of the research, the expectations and the risks. Every participant had the right to withdraw their consent at any time.

**Conclusion**

Presented here is an evaluation of the methods used in exploring rural patient perceptions of SCN as they stay away from home for RT. The study aimed to discover an area of SCN few have researched previously. The nature and approach of the research has influenced the chosen methods of sampling, data collection and analysis. The interpretation of the lived experience of participants went through rigorous, systematic and focused IPA. Generating good data relies on good methodology, yet performing good analysis relies on more than following a rigorous process. The research outcomes must make sense to the phenomenon being studied. Intrinsic connections were made within the data and to the world outside of that data, through social constructs and contextual relationships, existing literature and theory and professional goals for effective patient-centred care.

**References**

(1) Rehman, A. A., & Alharthi, K. (2016). An introduction to research paradigms. *International Journal of Educational Investigations,* 3(8), 51-59.

# (2) Green, J., & Thorogood, N. (2014). *Qualitative methods for health research*(Third edition.). Los Angeles, California: SAGE.

(3) Whelan, T.J., Mohide, E. A., Willan, A.R, Arnold, A., Tew, M., Sellick, S., Gafni, A., & Levine, M.N. (1997). [The supportive care needs of newly diagnosed cancer patients attending a regional cancer center](https://shu.primo.exlibrisgroup.com/discovery/fulldisplay?docid=gale_ofa19987848&context=PC&vid=44SHU_INST:44SHU_VU1&lang=en&search_scope=MyInst_and_CI&adaptor=Primo%252525252520Central&tab=Everything&query=any,contains,Supportive%252525252520Care%252525252520Needs%252525252520cancer%252525252520interview&facet=citedby,exact,2017204086360149473). *Cancer*, 80, 1518–24

(4) Bury, M. (1986). Social constructionism and the development of medical sociology. *Sociology of Health and Illness*, 8, 137–69.

(5) Rogers-Clark, C. (2002). Living with breast cancer: The influence of rurality on women’s suffering and resilience. A postmodern feminist inquiry. *Australian Journal of Advanced Nursing, 20*(2), 34–39.

(6) Loughery, J & Woodgate, R.I. (2015) Supportive care needs of rural individuals living with cancer: A literature review. *Canadian Oncology Nursing Journal*. 25(2), 2368-8076

# (7) Tod, A. (2015) Interviewing. *The Research Practice in Nursing*, 7^th^ Ed. Edited by Kate Gerrish and Judith Lthlean. John Wiley & Sons Ltd.

(8) McElduff, P., Boyes, A., Zucca, A., & Girgis, A. (2004). *Supportive Care Needs Survey: A guide to administration, scoring and analysis*. Centre for Health Research & Psychology (CHeRP).

# (9) Brown, N.M.K., Lui, C., Robinson, P.C., & Boyle, F.M. (2015). Supportive care needs and preferences of lung cancer patients: a semi-structured qualitative interview study. *Support Care Cancer* 23, 1533–1539.

(10) Moser A, Korstjens I. Series: Practical guidance to qualitative research. Part 3: Sampling, data collection and analysis. *Eur J Gen Pract*. 2018;24(1):9-18.

# (11) Reid, K., Flowers, P., & Larkin, M. (2005). Exploring lived Experience. *The Psychologist* 18, 18-23.

(12) Wilkes, L.M., White, K., Mohan, S., & Beale, B. (2006). Accessing metropolitan cancer care services: Practical needs of rural families. *Journal of Psychosocial Oncology*, 24(2), 85–101.

(13) Duggleby, W.D., Penz, K.L., Goodridge, D.M., Wilson, D.M., Leipert, B.D., Berry, P.H., & Justice, C.J. (2010). The transition experience of rural older persons with advanced cancer and their families: A grounded theory study. *BMC Palliative Care*, 9(5), 1–9.

(14) Edwards, L.B., & Greeff, L.E. (2018) Evidence-based feedback about emotional cancer challenges experienced in South Africa: A qualitative analysis of 316 photo-voice interviews, *Global Public Health*, 13(10), 1409-1421.

# (15) NCPHU, North Coast Public Health Unit. (2015). NNLHD Our Population Factsheet. Retrieved from https://nnswlhd.health.nsw.gov.au/wp-content/uploads/NNLHD_our_population_factsheet_160208.pdf

(16) Clavarino, A. M., Lowe, J.B., Carmont, S.A., & Balanda, K. (2002). The needs of cancer patients and their families from rural and remote areas of Queensland. *Australian Journal of Rural Health*.10(4), 188-95.

(17) Fitch, M.I., Gray, R.E., McGowan, T., Bunskill, I., Steggles, S., Sellick, S., Bezjak, A., & McLeese, D. (2003). Traveling for radiation cancer treatment: Patient perspectives. *Psycho-Oncology*, 12(7), 664–674.

(18) Ricoeur, P. (1976). *Interpretation theory: Discourse and the surplus of meaning* (5th ed). Fort Worth: Texas Christian University Press.

(19) Newton, B., Rothlingova, Z., Gutteridge, R., Lemarchand, K., & Raphael, J. (2012). No room for reflexivity? Critical reflections following a systematic review of qualitative research. *Journal of Health Psychology*, *17*(6), 866–885.

(20) Jumisko, E., Lexell, J. & Söderberg, S. (2007) [Living With Moderate or Severe Traumatic Brain Injury: The Meaning of Family Members' Experiences,](http://www.brown.uk.com/teaching/qualitativepostgrad/jumisko.pdf) *Journal of Family Nursing,* 13, 353-369.

# (21) Muntlin, A.A., Brovall, M., Wengstrom, Y., Conroy, T., & Kitson, A.L. (2018). Descriptions of fundamental care needs in cancer care—An exploratory study. Journal of Clinical Nursing, 27(11-12), 2322-2332.

(22) Okuhara, T., Ishikawa, H., Urakubo, A., Hayakawa, M., Yamaki, C., Takayama, T., & Kiuchi, T. (2018). Cancer information needs according to cancer type: A content analysis of data from Japan's largest cancer information website. *Preventive medicine reports*, *12*, 245–252.

(23) Soanes, L., & Gibson, F. (2018). Protecting an adult identity: A grounded theory of supportive care for young adults recently diagnosed with cancer. *International Journal of Nursing Studies*, *81*, 40–48.

(24) Alase, A. (2017). The Interpretative Phenomenological Analysis (IPA): A Guide to a Good Qualitative Research Approach. *International Journal of Education and Literacy Studies*, [S.l.], 5(2), pp. 9-19, Available at: <<https://www.journals.aiac.org.au/index.php/IJELS/article/view/3400>>. Date accessed: 20 oct. 2021.

(25) Lincoln YS, Guba EG. *Naturalistic inquiry*. Sage; 1985.

# (26) Ballinger, B. (2006). Demonstrating Rigour and Quality. In L. Finlay (Ed.), *Qualitative Research for Health Professionals: Challenging Choices (*pp. 235-246). Chichester, England: John Wiley & Sons.

(27) Tong, A., Sainsbury, P. & Craig, J (2001). Consolidated criteria for reporting qualitative research (COREQ): a 32-item checklist for interviews and focus groups, *International Journal for Quality in Health Care*, 19(6), pp.349–357

(28) Finlay, L., & Gough, B. (2003). *Reflexivity a practical guide for researchers in health and social sciences*. Oxford: Blackwell Science.

(29) LeVasseur, J. (2003). The Problem of Bracketing in Phenomenology, *Qualitative Health Research 13(3)*, 408–20.

(30) Fischer, C.T. (2008). Bracketing in qualitative research: Conceptual and practical matters. *Psychotherapy Research, 19(4-5),* 583-590.

(31) Tufford, L., & Newman, P. (2010). Bracketing in Qualitative Research*. Qualitative Social*. *11(1),* 80–96.

**Appendix 1:** Exert from the researcher’s reflexive journal

“Re-listening to interviews I realised that there is a lot more that was said to record and pick up on. For example in the first interview the patient had talked a lot more about them being to blame for the funny turn they had in thee department. They also talked about their journey to diagnosis and the importance the GP played in that. In the third interview the participant talked a lot about his values, and there was a lot I had not recorded about his views and opinions about life in general I had initially dismissed as unrelated to treatment or being away from home. So notes that I had made initially were largely superficial and did not link in some of the aspects that have later been highlighted by other participants. This was influenced by the bias to be practical and logically label in short bullets points. A deeper understanding takes time and a more thorough analysis.”

**Appendix 2:** Consolidated criteria for reporting qualitative studies (COREQ): 32-item checklist (27)

| **No** | **Item** | **Guide questions/description** |
| --- | --- | --- |
| **Domain 1: Research team and reflexivity** | | |
| Personal Characteristics | | |
| 1. | Interviewer/facilitator | The lead researcher conducted all interviews. |
| 2. | Credentials | The lead researcher has a background in psychology, after completing a degree in psychology in 2005; then working for the NHS in health promotion, she retrained as a Radiation Therapist in 2012, gaining her Post-graduate Diploma in Radiotherapy and Oncology in Practice with Sheffield Hallam University. She moved to Australia in 2016 to work as a Radiation Therapist. |
| 3. | Occupation | Radiation Therapist |
| 4. | Gender | Female |
| 5. | Experience and training | What experience or training did the researcher have? |
| Relationship with participants | | |
| 6. | Relationship established | No established relationship. But known as a healthcare worker because she was in uniform. |
| 7. | Participant knowledge of the interviewer | Has met a team of Radiation Therapists already (with the same uniform). They knew the researcher wanted to explore and understand patient experiences. |
| 8. | Interviewer characteristics | Proven ability to listen, knowledge of radiation therapy, cancer treatment and interest in research. |
| **Domain 2: study design** | | |
| Theoretical framework | | |
| 9. | Methodological orientation and Theory | Interpretive phenomenological approach. See above research paradigm section. |
| Participant selection | |  |
| 10. | Sampling | Typical case purposive sampling |
| 11. | Method of approach | Face-to-face invitation with written information provided, followed by a phone call or another face-to-face reminder. |
| 12. | Sample size | 13 |
| 13. | Non-participation | 5 potential participants declined the invitation to participate (not included in the total 13). Reasons included tiredness, practicalities or dislike talking about themselves. |
| Setting | |  |
| 14. | Setting of data collection | Face-to-face in a private clinical interview room with soft seating. |
| 15. | Presence of non-participants | Participants were made aware they could have family or carers with them but none of them requested this. |
| 16. | Description of sample | Invited participants met the following criteria:   - Currently having RT treatment > three weeks - Staying away from home but close to the treatment centre for > three days a week - Home address located > two hours round trip from the treatment centre - English speaking adults |
| Data collection | |  |
| 17. | Interview guide | See supporting information on interview guide. A pilot of the first 5 participants was conducted. |
| 18. | Repeat interviews | No. |
| 19. | Audio/visual recording | Audio |
| 20. | Field notes | Yes – notes made immediately after the interviews |
| 21. | Duration | 20-90 minutes |
| 22. | Data saturation | Yes - with the supporting investigator |
| 23. | Transcripts returned | No – notes were returned and checked for understanding. |
| **Domain 3: analysis and findings** | | |
| Data analysis | |  |
| 24. | Number of data coders | 1 |
| 25. | Description of the coding tree | No |
| 26. | Derivation of themes | No - exploratory research |
| 27. | Software | N/A |
| 28. | Participant checking | No |
| Reporting | |  |
| 29. | Quotations presented | Yes quotations were used but the participants were not given numbers or pseudonyms |
| 30. | Data and findings consistent | Yes |
| 31. | Clarity of major themes | Yes – with research supervisor |
| 32. | Clarity of minor themes | Yes - specific examples were presented and the individual circumstances were described, which enhanced the diversity of cases discussed. |
