# Supplementary material for: Being away from home for cancer treatment: a qualitative study of patient experience and supportive care needs during radiation therapy
Source: J Med Radiat Sci. 2022 Apr 4;69(3):336–47. doi: 10.1002/jmrs.578 (PMC9442298; doi:10.1002/jmrs.578)
Supplement: Supplementary file 3 — Appendix S3. Interview guide. [file JMRS-69-336-s001.docx]

**Supporting Information 3 - Interview Guide**

**Being away from home for cancer treatment: A qualitative study of patient experience and supportive care needs during radiation therapy**

Authors: Vanessa Knibbs ^1^, BScPsycMgt, PostGradDipRad; Stephen Manley ^1^, MBusAdmin, BApplSci-MedRad, DipProjMgt, GradCertBus

Affiliations: ^1^ North Coast Cancer Institute, Lismore, Northern NSW LHD.

Corresponding Author Address: [ness_knibbs@yahoo.co.uk](mailto:ness_knibbs@yahoo.co.uk)

Can you tell me about your experiences of living in a rural or remote area and attending for cancer treatment here?

Prompts

- Can you tell me a bit about your living circumstances in a rural area and how it impacts on you as a cancer patient getting radiation therapy treatment here?
- How do you find travelling to and from the treatment centre in regard to time and cost?
- How do you feel about the distance you have to travel the cancer centre?
- How do you make the journey to the cancer centre?
- Who do you travel with?
- What do you think about staying locally for the treatment?
- What do you think about the coordination of appointments?

Can you tell me about the information health care professionals have given you about your treatment as a rural cancer patient attending treatment away from home?

Prompts

- How do you feel about the accuracy of the information provided to you?
- How much information were you given about the options for staying locally or travelling?
- How well informed do you feel as a cancer patient living in a rural location?
- Are you aware of any support groups (face to face or online) available to you?

How do you feel about the support you have going through radiation therapy treatment away from home at the moment?

Prompts

- How well supported do you feel physically?
- How do you feel about being able to undertake your normal daily activities?
- Can you tell me about any feelings of uncertainty or worry you may have now, or have had in the past, about attending for treatment away from home?
- In general, how do you feel about the support you have received from your health care providers?
- How well supported do you feel emotionally?
- Are there any improvements that might be made that could have enhanced the care you received so far?

How well do you feel that you and your family are able to cope during radiation therapy treatment away from your home?

Prompts

- As a patient living in a rural community can you tell me about the social and psychological support you have?
- How well supported do you feel socially?
- How do you feel about the support you have from your family or friends during treatment, here at the centre and at home in your community?
- How supported do you feel performing activities such as attending appointments, picking up prescriptions or buying food?
- How do you feel about the support you have to cope with the physical challenges of the treatment such as skin reactions or decreased mobility?

What challenges, if any, do you find are associated with being geographically far away from the health services?

Prompts

- Can you tell me about the practicalities you experience such as getting to appointments on time, making sure you eat well during treatment?
- If you cannot identify any challenges, can you talk to me about any positive aspects to being from a rural area?
- Can you tell me about any anxiety or fear of travelling to/from the treatment centre you may have?
- What are your thoughts about returning to a rural or remote area after treatment has finished?
- Can you tell me about how being away from home for treatment may affect your close relationships?

How do you think health professionals can better meet your needs as a rural patient getting radiation therapy treatment here?

Prompts

- Tell me more about your interactions with health care professionals as a patient attending for cancer treatment away from home.
- How do you feel your supportive care needs can be met better during treatment for cancer away from home?
- Can you tell me about any worries or concerns you have about your health care needs?
